# Comprehensive analysis of classroom microclimate in context to health-related national and international indoor air quality standards

**DOI:** 10.3389/fpubh.2024.1440376

**Published:** 2024-08-12

**Authors:** Tudor Caciora, Alexandru Ilieş, Zharas Berdenov, Hadeel Sa'ad Al-Hyari, Dorina Camelia Ilieş, Bahodirhon Safarov, Thowayeb H. Hassan, Grigore Vasile Herman, Nicolaie Hodor, Bahadur Bilalov, Ana Cornelia Peres

**Affiliations:** ^1^Department of Geography, Tourism and Territorial Planning, Faculty of Geography, Tourism and Sport, University of Oradea, Oradea, Romania; ^2^Faculty of Science, L.N. Gumilyov Eurasian National University, Nur-Sultan, Kazakhstan; ^3^School of Business Administration, Al-Balqa Applied University, Salt, Jordan; ^4^Department of Digital Economy, Samarkand State University, Samarkand, Uzbekistan; ^5^Social Studies Department, College of Arts, King Faisal University, Al Ahsa, Saudi Arabia; ^6^Tourism Studies Department, Faculty of Tourism and Hotel Management, Helwan University, Cairo, Egypt; ^7^Faculty of Geography, “Babes-Bolyai” University, Cluj-Napoca, Romania; ^8^Department of Tourism Business, Azerbaijan University of Tourism and Management, Baku, Azerbaijan; ^9^Faculty of Environmental Protection, University of Oradea, Oradea, Romania

**Keywords:** indoor air quality, environmental pollutants, student health, air pollution monitoring, public health

## Abstract

Indoor air quality (IAQ) and indoor air pollution are critical issues impacting urban environments, significantly affecting the quality of life. Nowadays, poor IAQ is linked to respiratory and cardiovascular diseases, allergic reactions, and cognitive impairments, particularly in settings like classrooms. Thus, this study investigates the impact of indoor environmental quality on student health in a university classroom over a year, using various sensors to measure 19 environmental parameters, including temperature, relative humidity, CO_2_, CO, volatile organic compounds (VOCs), particulate matter (PM), and other pollutants. Thus, the aim of the study is to analyze the implications of the indoor microclimate for the health of individuals working in the classroom, as well as its implications for educational outcomes. The data revealed frequent exceedances of international standards for formaldehyde (HCHO), VOC, PM_2.5_, NO, and NO_2_. HCHO and VOCs levels, often originating from building materials and classroom activities, were notably high. PM_2.5_ levels exceeded both annual and daily standards, while NO and NO_2_ levels, possibly influenced by inadequate ventilation, also surpassed recommended limits. Even though there were numerous exceedances of current international standards, the indoor microclimate quality index (IMQI) score indicated a generally good indoor environment, remaining mostly between 0 and 50 for this indicator. Additionally, analyses indicate a high probability that some indicators will exceed the current standards, and their values are expected to trend upwards in the future. The study highlighted the need for better ventilation and pollutant control in classrooms to ensure a healthy learning environment. Frequent exceedances of pollutant standards can suggest a significant impact on student health and academic performance. Thus, the present study underscored the importance of continuous monitoring and proactive measures to maintain optimal indoor air quality.

## 1 Introduction

Indoor Air Quality (IAQ) and Indoor Air Pollution (IAP) are fundamental interconnected problems that significantly impact the quality of life, particularly in urban environments (1). Poor IAQ is associated with various adverse health outcomes, including increased mortality and morbidity. The presence of indoor pollutants, which can originate from activities such as smoking, cooking, heating, and the use of household products, poses serious health risks. These pollutants contribute to conditions such as respiratory and cardiovascular diseases, allergic reactions, cancers, and specific syndromes like sick building syndrome and building-related illnesses (2–5).

Recent studies have highlighted the importance of effective IAQ monitoring and mitigation strategies to protect public health. Modern IAQ monitoring systems utilize wireless technologies and advanced sensors to provide real-time data on pollutant levels, allowing for timely interventions (6). Additionally, the development of novel materials for sensors and smart home technologies offers promising solutions for improving IAQ (7). For instance, research has shown that indoor pollutants like particulate matter (PM), CO, and VOC are significantly influenced by household activities and the type of fuel used for heating. These pollutants are linked to severe health issues such as chronic obstructive pulmonary disease and lung cancer, particularly in rural areas where coal burning is common, but not only (8).

Children, older adults, and those with pre-existing syndromes are especially vulnerable to the adverse effects of IAP, which can lead to respiratory problems and infections. This vulnerability is exacerbated in households with poor ventilation and high pollutant concentrations (9). Therefore, enhancing IAQ through effective monitoring and innovative mitigation strategies is crucial for reducing health risks and improving overall public health in urban environments.

Nowadays, IAQ in classrooms is a critical factor influencing the health and academic performance of students and teachers. Poor IAQ can lead to a range of adverse health effects, including respiratory and cardiovascular diseases, allergic reactions, and cognitive impairments, which can significantly impact learning outcomes and overall wellbeing (10–12). Studies have shown that classrooms often contain high levels of various air pollutants. These pollutants originate from outdoor sources, such as traffic emissions, and indoor activities, including the use of paints, markers, and cleaning products (13). Good IAQ in schools is essential to provide a safe, healthy, productive, and comfortable environment for students, teachers, and other school staff. Various air pollutants found in classrooms, sometimes even at low concentrations, can impact students' health, particularly respiratory health, classes attendance, and academic performance (14–16). For instance, higher levels of hazardous air pollutants have been associated with decreased concentration, productivity, and overall academic performance among university students (15). A study conducted in university classrooms revealed that inadequate ventilation and high levels of CO_2_, PM, and other pollutants significantly affect students' ability to focus and perform academic tasks (17). Other studies found that improvements in IAQ, such as through renovations, resulted in better standardized test scores and academic outcomes in a pre-university school, suggesting similar potential benefits in higher education environments (18).

The most common pollutants found in classrooms include PM, VOC, CO, and biological contaminants like mold and bacteria. Particulate matter, including PM_10_ and PM_2.5_, originates from outdoor sources such as traffic emissions and indoor sources like dust and classroom activities (19, 20). Various research has shown that high levels of PM in classrooms are associated with adverse health effects, including respiratory symptoms, and reduced cognitive performance (14, 21). VOCs are emitted from sources like paints, cleaning supplies, and building materials, causing health issues such as irritation, headaches, and chronic effects like liver and kidney damage. Poor ventilation exacerbates VOC accumulation, increasing the risk for students and staff (22, 23). CO_2_ levels, due to inadequate ventilation and high occupancy, indicate poor air quality, leading to symptoms like headaches, dizziness, and reduced cognitive function (24, 25). Biological contaminants like mold, bacteria, and viruses thrive in classrooms with poor humidity control and inadequate cleaning, leading to allergies, respiratory issues, and infections. Low concentrations of H_2_S cause eye irritation and nausea, and high concentrations can induce fainting and death (26). SO_2_ is associated with increased respiratory symptoms and premature death (27, 28), at the same time O_3_, NO, and NO_2_ irritate the airways and can cause severe cardiopulmonary problems and death (29, 30). Molds can grow on damp surfaces, releasing spores that exacerbate asthma and other respiratory conditions (31).

Based on those mentioned above, the present study aims to monitor and evaluate the internal microclimate of a classroom within the Faculty of Geography, Tourism, and Sport (FGTS) at the University of Oradea, Romania, with the objective of determining the influence of specific microclimate parameters such as temperature (T), relative humidity (RH), CO, and other pollutants on human health, wellbeing, concentration levels, and the academic performance of students. Using various environmental indicators, alongside advanced statistical techniques, this research offers a thorough understanding of the impact of these variables on human health and wellbeing. The outcomes will enrich the discourse on IAQ within the classroom, thereby harmonizing our methodologies with international standards to achieve a balanced approach between the preservation of human health and the need for a clean environment conducive to scientific endeavors.

## 2 Materials and methods

The monitored classroom is an IT-dedicated room, containing 16 computers and having a volume of 441 m^3^. The room can simultaneously accommodate 20 students and one teacher, and it is used intermittently during the academic year from Monday to Friday between 8 am and 8 pm. Approximately 302 FGTS students used the classroom over the monitoring period, with an average activity period for a student being between 2 and 4 h per week, and for a teacher between 2 and 10 h.

The selection of this specific classroom was based on its representativeness of modern educational environments, including computers, various usage patterns, and the need to monitor a comprehensive set of environmental parameters over a full academic year. At the same time, IT classrooms are equipped with multiple computers and other electronic equipment that can emit volatile chemicals and thermal radiation, which can influence IAQ, and the classes taught here require a high degree of concentration and cognitive performance, making the learning environment particularly important.

The internal microclimate is controlled by HVAC systems throughout the year, but these may be insufficient or non-compliant when many students are present simultaneously in the space. Oradea, the municipality where the University of Oradea is located, experiences a temperate-continental climate, characterized by hot summers and cold winters. This climatic context can influence indoor air quality, as HVAC systems need to adjust to significant temperature variations and maintain consistent indoor conditions despite external weather fluctuations.

### 2.1 Analysis of pollutants variations inside the classroom

**Figure 1:**
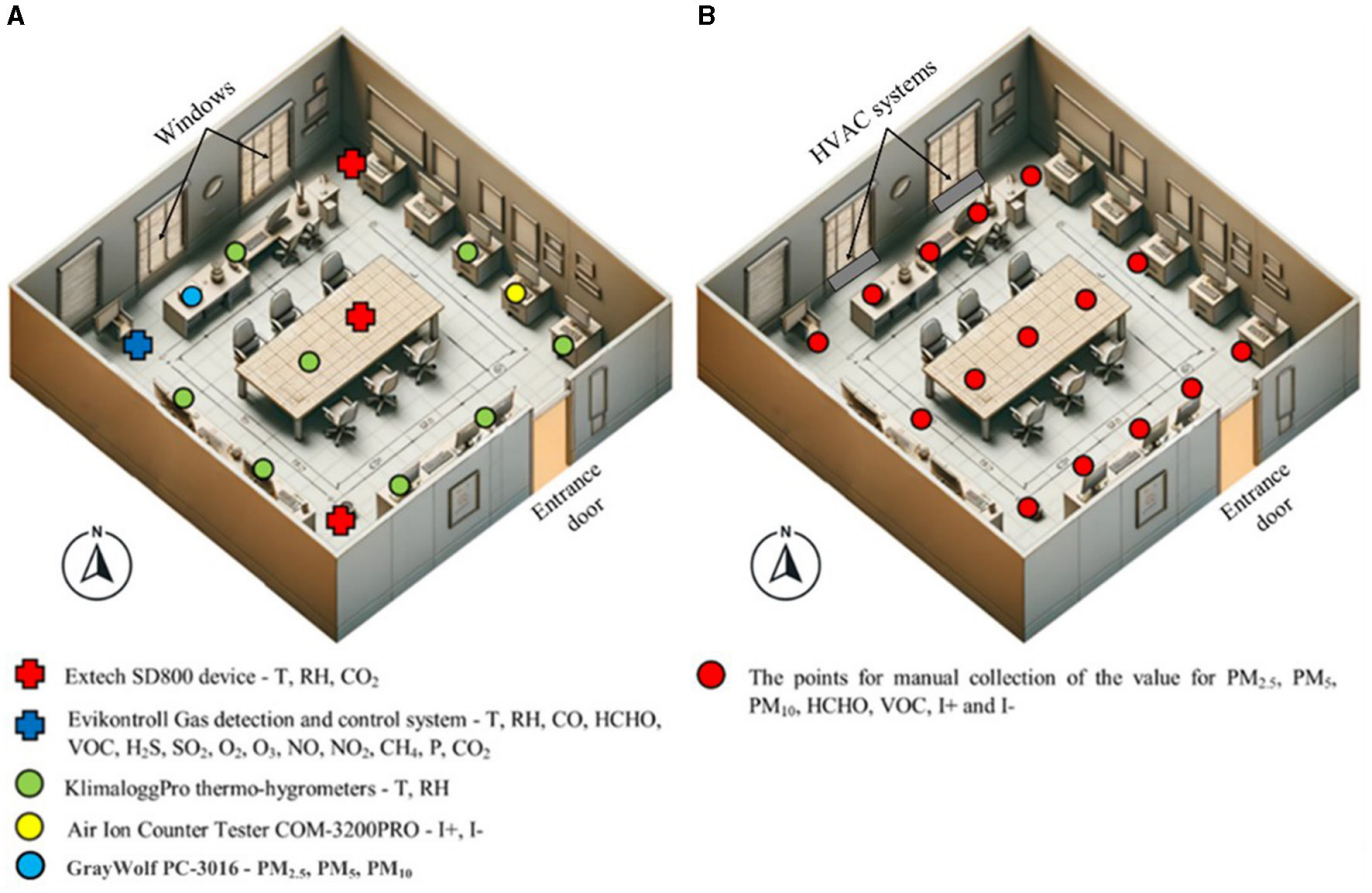
The distribution at the level of the classroom of the sensors for monitoring the indoor microclimate **(A)** and the manual data collection points **(B)**.

Pollution monitoring was carried out continuously during 1 calendar year, between September 1, 2022, and August 31, 2023. During this period, a number of 19 indicators were monitored, as follows: T, RH, pressure (P), CO_2_, CO, HCHO, VOC, H_2_S, SO_2_, O_2_, O_3_, NO, NO_2_, CH_4_, PM_2.5_, PM_5_, PM_10_, the concentration of positive ions (I+), and negative ions (I-). For most of the parameters, the use of datalogger sensors programmed to record and store the data every minute was considered, in order to then realize hourly averages for the targeted indicators. They were distributed throughout the room to cover it as well as possible, determining the values of the analyzed indicators with high accuracy (Figure 1A). For other indicators (I+, I-, and PM in particular), manual measurements were made before classes, during and after them, and for other indicators, additional manual measurements were made (if necessary) during the classes, such as VOC and HCHO. All the sensors used were chosen in such a way as to determine the indicators with the highest possible accuracy (Table 1). The results obtained were reported to the international standards in force for the targeted parameters concerning their influence on human health and wellbeing.

**Table 1:** Technical information about the sensors used to monitor the indicators inside the classroom.

**Sensor**	**Producer**	**Pollutants measured**	**Accuracy**	**No. of sensors used**	**Datalogger**
Extech SD800 devices	Extech Instruments, Nashua, NH, USA	T, RH, CO_2_	±0.8°C (T), ±4% (RH), ±40 ppm (CO_2_)	3	✓
Evikontroll Gas detection and control system devices	Evikontroll Gas, Tartu, Estonia	T, RH, CO_2_, CO, HCHO, VOC, H_2_S, SO_2_, O_2_, O_3_, NO, NO_2_, CH_4_, P	±0.5°C (T), ±5% (RH), ±50 ppm (CO_2_), ±1 ppm (CO), ±0.01 ppm (HCHO, VOC), ±0.1 ppm (H_2_S, SO_2_, O_3_, NO, NO_2_), ±0.01% (O_2_, CH_4_), ±1 hPa (P)	2	✓
KlimaloggPro thermo-hygrometers	TFA, Ottersberg, Germany	T, RH	±1°C (T), ±3% (RH)	8	✓
BLATN BR-smart-123s device	BLATN Science and Technology, Beijing, China	HCHO, VOC	±5% (HCHO, VOC)	1	×
Air Ion Counter Tester COM-3200PRO II	Universal Plan Co., Tokyo, Japan	I+, I-	±10 ions/cm^3^ (I+, I-)	1	✓
PCE-PCO 2	PCE Instruments UK, Southampton, UK	PM_2.5_, PM_5_, PM_10_	up to ±5% (PM_2.5_, PM_5_, PM_10_)	1	×
GrayWolf PC-3016	GrayWolf Sensing Solutions, Shelton, Connecticut, USA	PM_2.5_, PM_5_, PM_10_	up to ±5% (PM_2.5_, PM_5_, PM_10_)	1	✓

The manually determined parameters (PM, I+, and I-) were measured at 15 points and uniformly distributed in the classroom in order to have the best possible distribution of pollutants at the room level (Figure 1B). The location of the collection points allows spatial analysis by means of cartograms that illustrate the distribution and evolution of these indicators in the analyzed room. The measurements were made at a height of 1.2–1.3 m, equivalent to the height of a sitting person of average height, to allow the analysis of the possible negative impact of these indicators on the health and wellbeing of the students. Regarding the manual PM determinations, the sampling times were 30 s for each collection point, considering the highest value measured for the sampling time.

Sensor calibration was implemented only for certain sensors, as others come pre-calibrated from the factory and do not require further user intervention. For those requiring calibration, it was conducted in controlled environments to minimize external influences, where the exact values of the respective indicators were known. Sensors were validated through cross-referencing and comparison with manual measurements. Additionally, continuous monitoring using dataloggers helped identify anomalies and validate data. Regular checks ensured that real-time data were accurate.

The collected data were analyzed both with reference to individual, hourly, daily, weekly, monthly, and annual values, depending on the need. R version 4.3.2, ArcMap 10.8.2, and MATLAB 9.7 software were used in the data analysis and interpretation process. At the same time, the recorded data were the basis for the realization of risk indices on human health and analyses regarding the probability that the recorded values will be exceeded in the future or not. All the indexes created were based on the thresholds in force accepted by the international bodies regarding the internal microclimate indicators considered.

### 2.2 Assessing the impact of the internal microclimate on human health

In order to assess the impact of indoor microclimate on human health, a simplified index designed to reflect IAQ was developed. This index aims to quantify internal environmental effects by comparing the average values of various selected indicators with existing international standards. The aim is to provide a clear understanding of internal environmental conditions and to facilitate the assessment of potential health risks. In the specialized literature, there are numerous studies that consider the creation of similar indices for the evaluation of the IAQ and its impact on human health, among which Saad et al. (32), Wagdi et al. (33), or Dionova et al. (34) are worth mentioning. However, in the case of the present study, an adaptation of it was chosen to reflect the needs of the study.

The following section details the mathematical formula of the indoor microclimate quality index (IMQI) (Equation 1), highlighting how these values are integrated and related to international air quality standards:


(1) $$\begin{array}{l} IMQI=\frac{\sum_{i=1}^{n}\left(Score_{i}\times z_{i}\right)}{\sum_{i=1}^{n}z_{i}} \end{array}$$

where *n* is the number of parameters considered, *Score*_*i*_ is the score for each parameter *i*, calculated according to the threshold or ideal interval, *z*_*i*_ is the weight assigned to each parameter.

The score component represents a parameter that considers the calculation of the ratio between the measured value of an indicator and its international standard in force. If the value is equal to or lower than the standard, the score is 0, indicating an ideal situation and a healthy internal microclimate. If the value exceeds the standard, the score increases proportionally to the exceedance and indicates a low quality of the internal microclimate. The score component (*S*_*i*_) is calculated based on the Equation 2:


(2) $$\begin{array}{l} S_{i}\left(\frac{MV-SV}{SV}\right)\;\times 100 \end{array}$$

where *MV* represents the measured value of indicator *i*, and *SV* represents the standard value of indicator *i*.

If an indicator does not have a fixed threshold, but falls within a well-established range, as is the case of O_2_, which according to international standards (35), must fall between a concentration of 19.5 and 23.5%, *S*_*O*2_ (score for the indicator O_2_) will be assigned as follows (Equations 3–5):


(3) $$\begin{array}{l} S_{O2}=0\;if\;19.5\%<\;MV_{O2}<23.5\% \end{array}$$

(4) $$\begin{array}{l} S_{O2\;}=\left(\frac{19.5\%-MV_{O2}}{19.5\%}\times 100\right)\;if\;O_{2}<19.5\% \end{array}$$

(5) $$\begin{array}{l} S_{O2\;}=\left(\frac{MV_{O2}-23.5\%}{23.5\%}\times 100\right)\;if\;O_{2}>23.5\% \end{array}$$

where *MV*_*O*2_ is the measured value for the O_2_ indicator. If the O_2_ level is between 19.5 and 23.5%, the score is perfect (0), if the O_2_ level is below 19.5%, the score increases proportionally with the exceeding of the lower limit, and if the O_2_ level is above 23.5%, the score increases proportionally with the exceeding of the upper limit.

The *z*_*i*_ component represents a significant weight assigned to each indicator. Thus, if a certain pollutant has a greater effect on people's health or comfort, it will be assigned a higher weighting factor in the model. By adjusting these factors, the model can balance the contributions of different variables to reflect their relative risk or importance more accurately to air quality. The specific interval for the *z*_*i*_ weight was established to be between 0 and 1 (on four levels), where 0 means no influence, and 1 means maximum influence. The weight was assigned in such a way to reflect the comparative importance of each pollutant in affecting IAQ and human health, considering the importance in the indoor environment, the impact on health, international standards and recommendations, scientific research in the field, the consensus of experts, etc.

**Figure 2:**
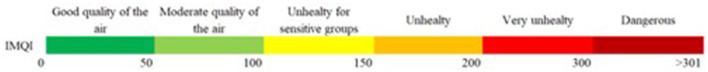
Classification of outdoor air quality according to the Environmental Protection Agency (34), based on an IMQI index similar to the one in the present manuscript.

A low IMQI score indicates good IAQ, which means that pollutant concentrations are below the thresholds set by international standards, and the indoor environment is considered healthy and safe for occupants. At the same time, a high score indicates a low quality of the indoor microclimate, indicating that one or more pollutants exceed the limits of international standards. Considering that there is no internationally accepted scale to classify IAQ using IMQI, in this model, we have chosen to report the results using the scale proposed by EPA (36) for air evaluation. Within this system of categorizing the degree of risk to human health of the outdoor air, the EPA considers its categorization on six different levels, from a good indoor microclimate quality (0–50) to a hazardous indoor environment for human health (over 301; Figure 2).

The use of the proposed US EPA model for outdoor conditions under indoor environmental conditions may be limited due to significant differences in pollution sources, dispersion patterns, and indoor environmental conditions, as well as limited data collection and confounding factors specific to the indoor environment, such as building materials, furniture, and human activities. However, the application of this model is justified as it provides a standardized and rigorous framework for air quality assessment and the establishment of benchmarks for the protection of human health in indoor environments in the absence of dedicated standards.

### 2.3 Statistical analysis of probabilities

To identify microclimatic patterns and fluctuations and to obtain a comprehensive understanding of thermal comfort conditions and air quality in the classroom, the determination of the empirical distribution function (EDF) was also considered to calculate the probability that a random value is more or less than or equal to a specific value. This function was calculated for each microclimate variable that exceeded the international standards in force (the standard being regarded as the threshold that will or will not be exceeded in the future). The EDF is used to evaluate the statistical behavior of the internal microclimate, including the determination of the median, percentiles, and variability (37, 38).

The specific formula for calculating the EDF is:


(6) $$\begin{array}{l} F\left(k\right)=\pi \frac{k-a}{n-2a+1}\;where\;0\;\leq a\leq 0.5 \end{array}$$

where *F(k)* is the probability of observing a value ≤ *k, a* is a constant that adjusts the formula, which must be between 0 and 0.5 and *n* is the sample size or the number of observations. The formula essentially normalizes the position of the value *k* within the observed data range, adjusted by *a*. The choice of *a* affects the weighting of the tails of the distribution (Equation 6).

The ARIMA model was also applied to analyze and predict the evolution of pollutant levels in the classroom. This was done to determine to what extent the pollution that exceeded the limits imposed by the international standards in force follows a certain trend or not, having the potential to increase in terms of concentrations in the future. The ARIMA model combines three main components: the autoregressive, integration, and moving average processes, each of which has an essential role in modeling and understanding the time series dynamics of pollutant concentrations.

Autoregressive processes of *p* order are described by the formula:


(7) $$\begin{array}{l} X_{t}=c+\emptyset_{1}X_{t-1}+\emptyset_{2}X_{t-2}+\ldots +\emptyset_{p}X_{t-p}+\varepsilon_{t} \end{array}$$

where *X*_*t*_ is the value of the time series at time *t, c* is the constant, Ø_1_*,...,Ø*_*p*_ are the coefficients of the autoregressive process, and ε_*t*_ represents the error term (39) (Equation 7).

The integration component of *d* order involves differentiating the time series to ensure its stationarity, being expressed by:


(8) $$\begin{array}{l} \nabla^{d}X_{t}={\left(1-L\right)}^{d}X_{t} \end{array}$$

where ∇^*d*^ denotes the differentiation operator of *d* order, and *L* is the delay operator (Equation 8).

The moving average processes of *q* order are defined as:


(9) $$\begin{array}{l} X_{t}=\mu +\varepsilon_{t}+\theta_{1}\varepsilon_{t-1}+\theta_{2}\varepsilon_{t-2}+\ldots +\theta_{p}\varepsilon_{t-p} \end{array}$$

where μ is the mean of the series, and θ_1_*,…*,θ_*p*_ are the coefficients that measure the influence of previous errors on the current value (40) (Equation 9).

Combining these components, the ARIMA model is expressed by the Equation 10:


(10) $$\begin{array}{l} ARIMA=\left(1-\overset{p}{\underset{i=1}{\sum }}\emptyset_{i}L^{i}\right){\left(1-L\right)}^{d}X_{t}=\left(1+\overset{q}{\underset{j=1}{\sum }}\emptyset_{j}L^{j}\right)\varepsilon_{t} \end{array}$$

In order to ensure the quality of the obtained results, taking into account the fact that for the ARIMA model the data series must be stationary, the null hypothesis of non-stationarity was tested for each data series (where a *p*-value < 0.05 indicated stationarity). For non-stationary series, differentiation was applied. The order of differentiation (d) was determined by the number of times differentiation to achieve stationarity. The autoregressive order function (p) was determined using the partial autocorrelation function, while the moving average order (q) was determined using the plot autocorrelation function. After model fitting, residuals were analyzed to ensure they resembled white noise. The absence of significant autocorrelations in the plots indicates that the residuals are white noise, suggesting a good model fit.

This approach allows us to analyze how past time series values and recorded errors affect the current value and make predictions about the future evolution of pollutants in the classroom, determining whether they will increase, decrease, or remain constant in the near future. This can be implemented even if the indoor microclimate in the classroom is mostly controlled with HVAC systems, because ARIMA can identify patterns and fluctuations that can be influenced by different factors, including the operation of this systems (41, 42).

## 3 Results

### 3.1 Analysis of pollution variations inside the classroom

The values obtained for the analyzed indicators were reported to multi-annual average standards, daily average standards and respectively to short-term exposure limit that can represent an immediate threat to life or health. The results indicate that only five pollutants (HCHO, VOC, NO, NO_2_, and PM_2.5_) exceeded the multi-year average values allowed by the international standards in force. At the same time, some exceedances of the daily values were also recorded for other indicators (T, RH, and O_3_), but these are small and only sporadic. Regarding short-term and very short-term exceeding of the limits, they were outlined only in the case of two indicators (HCHO and PM_2.5_), the values being still quite small (Table 2).

**Table 2:** The values obtained for the analyzed indicators, being reported to the international standards in force.

**Pollutant**	**MU**	**Max**	**Min**	**Avg**	**Std. dev**.	**MAA**	**PE—MAA**	**DA**	**PE—DA**	**STEL/IDLH**	**PE—STEL/IDLH**
T	°C	27.1	18.1	22.9	2.1	20–24 (43)	14	18–26 (44, 45)	0.2	×	×
RH	%	60.6	17.6	38.5	10.2	30–50 (46)	28.5	30–70 (47)	22.9	×	×
CO_2_	Ppm	2,744	450	633.8	176.4	1,000 (48)	4.1	5,000 (49)	0	10.000–50.000 (50)	0
HCHO	mg/m^3^	0.082	0	0.057	0.063	0.04 (51)	58.5	0.05 (47)	49.4	0.100 (52)	5.8
VOC	mg/m^3^	4.8	0	1.4	0.65	1 (53)	69.2	3 (54)	0.4	590 (54)	0
I+	no/cm^3^	2,200	200	799.5	358.5	<1,000 (55, 56)	25	×	×	×	×
I-	no/cm^3^	2,650	300	1,168.4	464.2	>1,000 (55, 56)	38.7	×	×	×	×
CO	mg/m^3^	5.60	0	1.79	0.74	×	×	10 (53)	0	30–40^a^	0
H_2_S	mg/m^3^	0.174	0.012	0.080	0.026	0.15 (53)	1	1.5 (53)	0	14^b^	0
SO_2_	μg/m3	106.7	7.9	46.2	18.6	50 (53)	42.5	125 (53)	0	13,000^a^	0
O_2_	%	22.74	20.39	21.67	0.5	×	×	19.5–23.5 (35)	0	10 (55)	0
O_3_	μg/m3	137.2	37.3	69	18.1	×	×	120	0.8	196^a^	0
NO	μg/m3	172.4	10.4	84.3	39.9	40 (53)	84.7	75 (53)	56.3	1,880^a^	0
NO_2_	μg/m3	260.9	36.9	107.4	41.2	40 (53)	99.5	75 (53)	78.8	1,880^a^	0
CH_4_	%	0.8	0.1	0.42	0.19	×	×	5 (57, 58)	0	15 (57, 58)	0
PM_2.5_	μg/m3	131.2	29.5	65.5	13.5	12 (59)	100	35 (59)	99.5	100 (60)	0.7
PM_5_	μg/m3	24.4	1.1	9.6	4	12 (59)	27	35 (59)	0	100 (60)	0
PM_10_	μg/m3	18.4	0.2	5.6	2.6	12 (59)	1	35 (59)	0	100 (60)	0
P	hPa	1,011.7	992.5	1,001.6	2.1	980–1,050 (61)	0	×	×	×	×

MAA, Multi-annual average standard values; PE—MAA, The percentage of exceeding the MAA; DA, Daily average standard values; PE—DA, The percentage of exceeding the DA; STEL/IDLH, Short-term exposure limit/Immediate threat to life or health standards; PE—STEL/IDLH, The percentage of exceeding the STEL/IDLH; Max, Maximum value during the entire monitoring period; Min, Minimum value during the entire monitoring period; Avg, Average value of the monitorings; Std. dev., Standard deviation; MU, Measure unit.

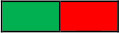
Does not exceed MMA/Exceed MMA.

^a^https://www.cdc.gov/niosh/index.htm (accessed January 8, 2024).

^b^https://eur-lex.europa.eu/LexUriServ/LexUriServ.do?uri=OJ:L:2009:338:0087:0089:EN:PDF (accessed May 28, 2024).

**Figure 3:**
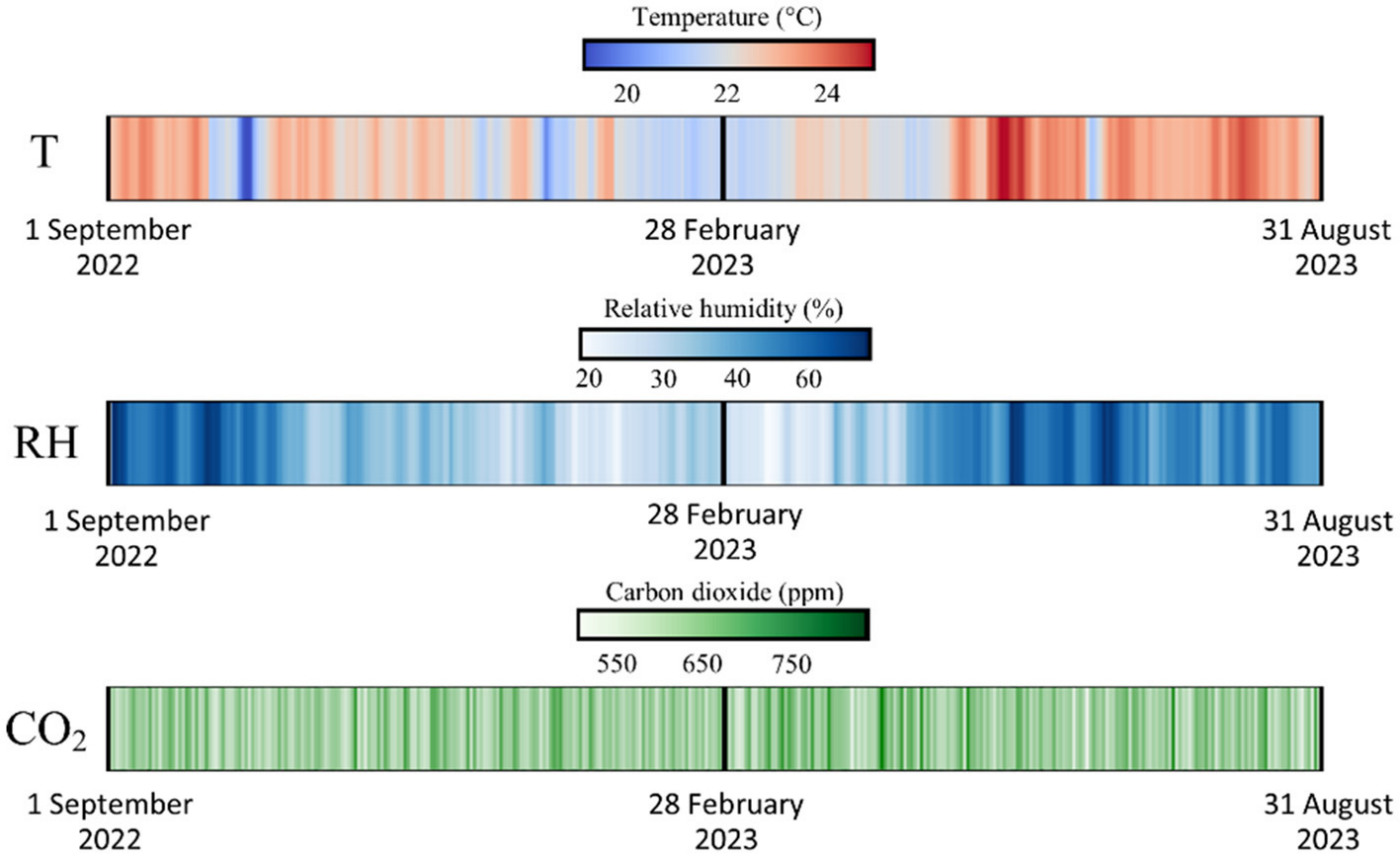
Variations of daily averages of T, RH, and CO_2_ inside the classroom analyzed during the period 01.09.2022–31.08.2023.

The average T recorded was 22.9°C, the maximum value being 27.1°C, and the minimum 18.1°C. These values indicate an average located in the range recommended by ASHRAE (20–24°C) for thermal comfort, but with significant deviations, suggesting that in some periods the conditions may be less comfortable (Figure 3). The RH average was 38.5% and varied between 17.60 and 60.60%. The average and most RH values are consistent within the EPA recommended range (30–50%) for minimizing risks related to mold growth and respiratory problems, although there are records exceeding the upper limit (Figure 3). According to the analysis performed on the data set, the average CO_2_ concentration was about 633.8 ppm. The maximum value recorded was 2,744.38 ppm, significantly above the threshold of 1,000 ppm recommended by the ANSI/ASHRAE standard for maintaining human health. However, ~95.9% of the CO_2_ records remained below the 1,000 ppm threshold, suggesting that air quality was adequate most of the time (Figure 3).

**Figure 4:**
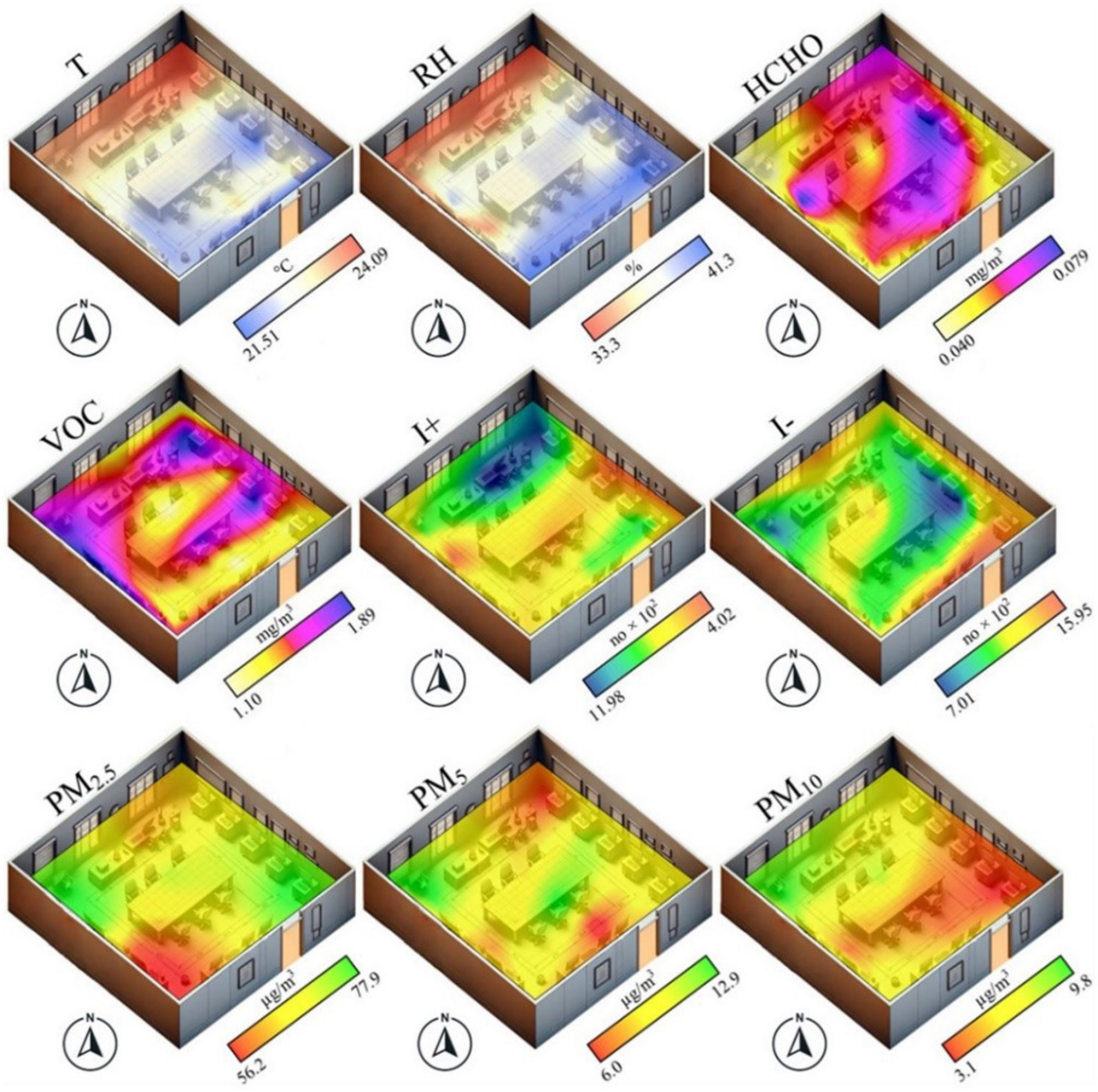
The spatial distribution at the classroom level of the average values of T, RH, HCHO, VOC, I+, I-, PM_2.5_, PM_5_, and PM_10_ (the values represent the multi-year average for each collection point).

The spatial distribution of T at the classroom level indicates a higher value in the immediate vicinity of the windows (24.09°C) and decreases with the distance from them and the proximity to the opposite wall (21.51°C). Regarding RH, its values are inversely proportional to those of T so that in the immediate vicinity of the windows, the lowest values are recorded (33.3%), while in the opposite area of the room, the values reach over 41% in the wettest points (Figure 4). This is also determined by the HVAC systems being located near the windows.

**Figure 5:**
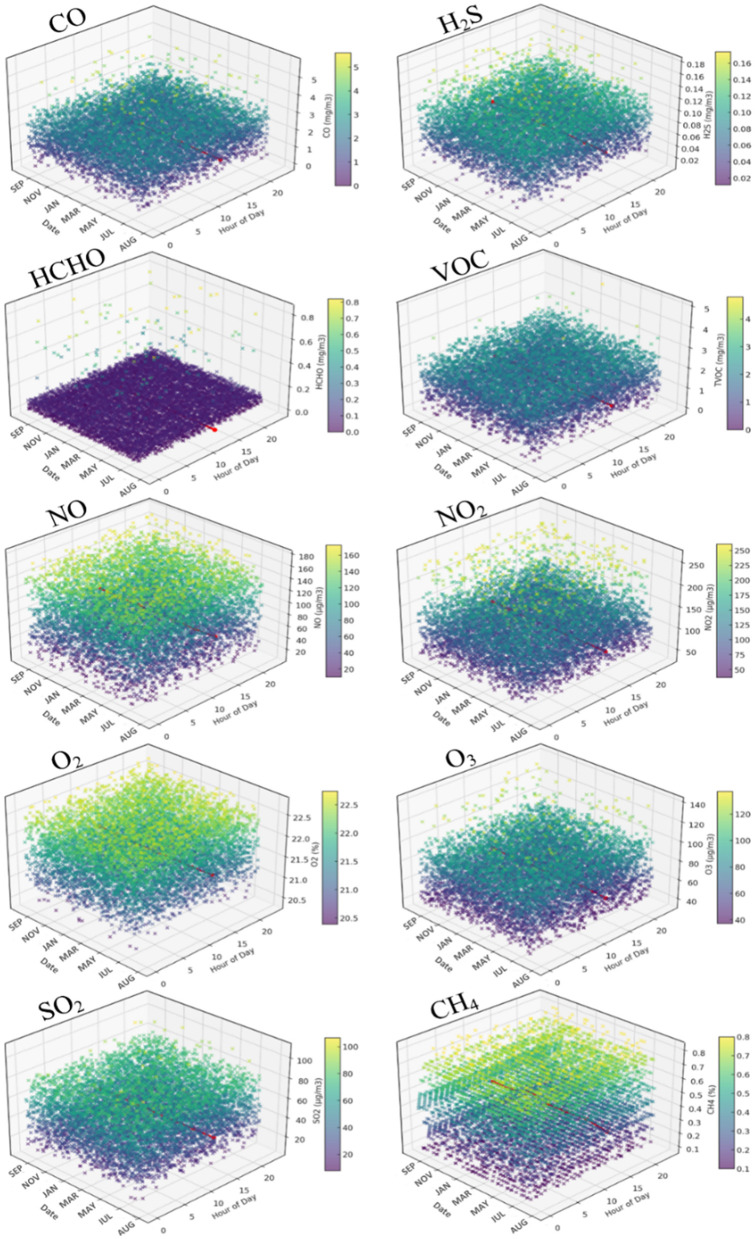
The values obtained by hours and days for the CO, H_2_S, HCHO, VOC, NO, NO_2_, O_2_, O_3_, SO_2_, and CH_4_ indicators in the case of the analyzed classroom.

The values obtained indicate an average VOC concentration of 1.43 mg/m3, exceeding the annual threshold of 1 mg/m3 (53), suggesting a possible influence on the health of the occupants. The variability of VOC indicates a maximum value of 4.8 mg/m3 and a minimum of 0.0 mg/m3, resulting in the daily limit of 3 mg/m3 being exceeded in only 0.4% of the measurements. In the northern area of the room, the VOC concentrations reached a maximum of 1.89 mg/m3, reflecting the uneven distribution inside the room (Figures 4, 5). The average concentration of HCHO was 0.057 mg/m3, a value above the daily threshold of 0.05 mg/m3, indicating a constant and potentially harmful exposure. Approximately 49.4% of the measurements exceeded the established daily limit, with a maximum value of 0.82 mg/m3. Regarding the spatial variation within the classroom, the high values (up to 0.079 mg/m^3^) are concentrated in the middle of the classroom, while on the sides (the exception being the window area), the values decrease to a minimum of 0.040 mg/m^3^ (Figure 4).

Average daily CO values were constant, remaining below the safety limit of 10 mg/m3, with a mean of 1.79 mg/m3 and variations between 0.0 and 5.6 mg/m3, indicating safe short- and long-term exposure. H_2_S concentrations varied daily with an average of 0.075 mg/m3 and daily maximum values of up to 0.174 mg/m3. Although 7% of days exceeded the daily threshold of 0.15 mg/m3, the overall values remained below the prolonged exposure threshold of 1.5 mg/m3. For SO_2_, average daily concentrations ranged between 12.29 and 103.03 μg/m3, with an annual average of 46.19 μg/m3, below the threshold of 50 μg/m3. Maximum daily values were below the 125 μg/m3 limit, indicating general compliance with international standards. O_2_ concentrations were maintained between 20.39 and 22.74%, with an average of 21.67%, according to international standards for IAQ. The average value of O_3_ was 68.98 μg/m3, below the threshold recommended by the World Health Organization (53). The values varied between 37.3 and 137.2 μg/m3, with the threshold of 120 μg/m3 being exceeded in 0.84% of the measurements. The average concentration of NO was 84.27 μg/m3, and that of NO_2_ was 107.40 μg/m3, with significant variations around the average and high frequencies of exceeding the international limits indicating the value of 75 μg/m3 as the daily average allowed. The average CH_4_ concentration was 0.57%, below the critical threshold, with values varying between 0.30 and 0.70%, demonstrating continuous compliance with environmental protection regulations (Table 2 and Figure 5).

**Figure 6:**
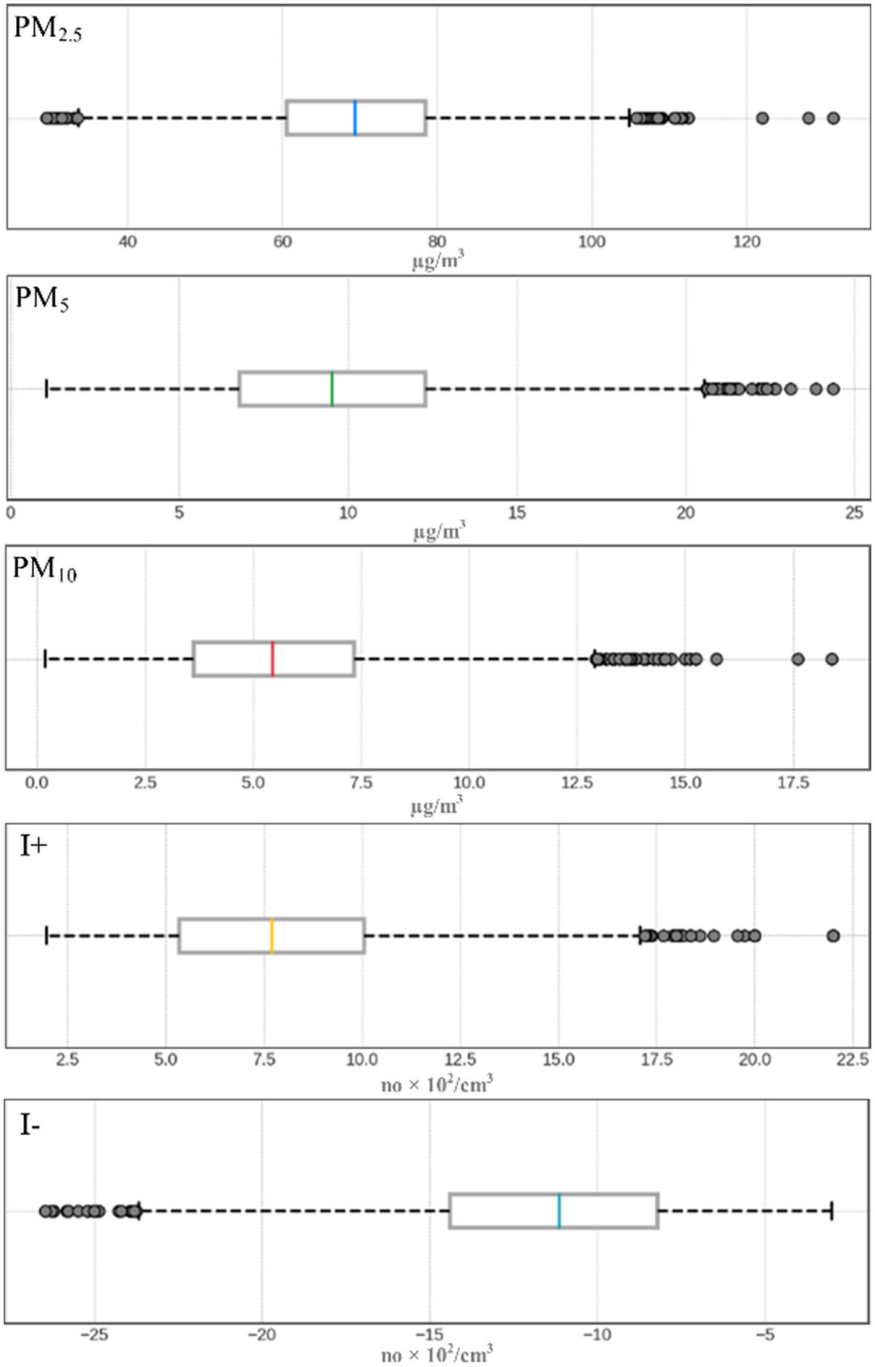
The concentration of PM_2.5_, PM_5_, PM_10_, I+, and I- in the classroom, during the period 01.09.2022–31.08.2023.

The average concentration of I+ was 799.5/cm^3^, with a maximum value of 2,200/cm^3^ and a minimum value of 200/cm^3^. Of the total recordings, ~25.4% exceeded the established threshold of 1,000/cm^3^, indicating a frequently high level of measurements exceeding the recommended threshold. On the other hand, the average concentration of I- was 1,168.4/cm^3^, with a maximum value of 2,650/cm^3^ and a minimum of 300/cm^3^. Of the total measurements, 61.3% exceeded the value of 1,000/cm^3^, considered to be conducive to the development of human activity, and 5.2% of the time, the values even exceeded 2,000/cm^3^ (Figure 6). Regarding the analysis of the spatial distribution of the two indicators at the level of the analyzed classroom, two different situations are individualized. In the case of I+, the area in the immediate vicinity of the windows obtained values higher than 1,000/cm^3^, while in the rest of the rooms the values do not exceed 800/cm^3^. I- is individualized by low values, up to 701/cm^3^, in the middle of the room, while the majority neighboring areas obtained values of over 1,000/cm^3^ (Figure 4). These results highlight, for the most part, a predominance of positive ions above the optimal level and an insufficiency of negative ions in relation to the values considered ideal for a healthy environment.

Regarding PM, the average concentration of PM_2.5_ was 69.51 μg/m3, with absolute values between 29.53 and 131.20 μg/m3. PM_5_ averaged 9.61 μg/m3, ranging between 1.06 and 24.37 μg/m3. PM_10_ recorded an average of 5.60 μg/m3, with absolute values between 0.18 and 18.40 μg/m3 (Figure 6). Thus, PM_2.5_ frequently exceeded the EPA annual standard (57) of 12 μg/m3, and 99.4% of the measurements exceeded the daily limit of 35 μg/m3, indicating persistent pollution. PM_5_ exceeded the annual threshold of 12 μg/m3 in 27.4% of cases without daily exceedances; however, PM_10_ exceeded the same threshold in only 1.1% of cases without exceeding the daily limit. These results suggest significant PM_2.5_ pollution, while PM_5_ and PM_10_ had rare exceedances of accepted standards.

Significant differences are observed when analyzing the spatial distribution of PM_2.5_, PM_5_, and PM_10_ in the classroom. PM_2.5_ shows lower values in the southern part (maximum of 56.2 μg/m3), while the rest of the hall registers values above 60 μg/m3, with maxima up to 77.9 μg/m3 in opposite corners of the room. The PM_5_ indicator has a variable distribution, with higher concentrations in the center and northwest of the hall and values below 8 μg/m3 in the surrounding areas. PM_10_ shows low values in the eastern and central part (minimum of 3.1 μg/m3), with higher concentrations near the windows (maximum of 9.8 μg/m3; Figure 4). High PM values in the vicinity of windows may be due to their opening for ventilation, allowing PM from outside to enter and increase indoor concentrations.

The results indicate significant PM_2.5_ pollution and frequent exceedances of acceptable concentrations for VOC, NO, NO_2_, and HCHO, suggesting a persistent negative influence on occupant health. Also, the spatial distribution of these pollutants reveals higher concentrations in certain classroom areas, especially near windows, highlighting the need to implement strict ventilation and pollution control measures to ensure a safe and healthy learning environment. The results are suggestive even if a US EPA standard designed for the outdoor environment was used to compare the results obtained. This is because there are no universally accepted standards for indoor PM_2.5_ and PM_10_ levels. The EPA standards are a benchmark because they are well-established and widely recognized for their health-based limits. The EPA's outdoor standards are based on extensive health research designed to protect public health, including sensitive populations. Using these standards as a reference for indoor air quality helps ensure that indoor environments are safe and healthy. Using the same standards for outdoor and indoor environments allows for easier comparison and understanding of air quality data. It provides a consistent framework for evaluating and managing air quality across different settings.

### 3.2 Assessing the impact of the internal microclimate on human health (IMQI)

To determine the IMQI, 18 indicators were used, considered to significantly impact human health if they are not mentioned in the accepted standards. Within this, it was considered to give a weight for each pollutant, depending on the impact that the pollutant has on health. Because there is no clear methodology for assigning weights and considering the fact that the WHO does not specify how they assign weights to pollutants, the indicator was assigned based on specialized literature (Table 3). At the same time, because people spend a limited amount of time inside the classroom, the standards to which the pollutant values were reported are those that aim at the daily variations allowed or the maximum values accepted for spending a limited amount of time in a certain indoor microclimate.

**Table 3:** The average obtained for each pollutant in the analyzed period, the value of the accepted international threshold at which it was reported, the unit of measure, the weight given to each indicator within the IMQI equation, and the scores obtained by the analyzed indicators.

**Pollutant**	**Pollutant average**	**International standards thresholds**	**Measure unit**	**Indicator weight**	**SLA**	**Score**
PM_2.5_	69.5	35	μg/m3	1	(62, 63)	98.6
PM_5_	9.6	35	μg/m3	1	(62, 63)	0
PM_10_	5.6	35	μg/m3	1	(62, 63)	0
O_3_	68.9	120	μg/m3	1	(64)	0
NO_2_	107.4	75	μg/m3	0.75	(65–67)	43.2
NO	84.3	75	μg/m3	0.75	(65–67)	12.4
SO_2_	46.2	125	μg/m3	0.75	(65)	0
CO	1.8	10	mg/m^3^	0.75	(62, 68)	0
VOC	1.4	3	mg/m^3^	0.75	(69)	0
HCHO	0.057	0.05	mg/m^3^	0.75	(69)	14
H_2_S	0.075	1.5	mg/m^3^	0.5	(70)	0
CO_2_	633.8	5,000	ppm	0.5	(70, 71)	0
CH_4_	0.42	5	%	0.25	(72)	0
O_2_	21.67	19.5–23.5	%	0.5	(70, 73)	0
T	22.88	20–24	°C	0.25	(74)	0
RH	38.5	30–50	%	0.25	(74)	0
I+	8 × 10^2^	<10 × 10^2^	No.	0.25	(75, 76)	0
I-	11.7 × 10^2^	>10 × 10^2^	No.	0.25	(75, 76)	0

^*^SLA—the specialized literature analyzed for the conferring of the indicator weights.

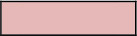
IMQI scores exceeding the ideal value (0).

Out of the 18 analyzed indicators, 14 obtained scores of 0, which indicates that the average values obtained for the entire monitoring period are lower than or equal to the international standards to which they were reported. The other four indicators recorded exceedances in a different quantity of the allowed thresholds. Thus, the smallest exceedances were consistent with NO (score 12.4), followed by HCHO (score 14) and NO_2_, with a score of 43.2. The most significant score was recorded in the case of the PM_2.5_ indicator, which was individualized by a coefficient of 98.6. Although most of the indicators comply with international standards, which indicates a generally satisfactory level of IMQ, high values in the case of some pollutants (especially PM_2.5_ and NO_2_) have the potential to represent a danger to human health, even acting in an individual way.

**Figure 7:**
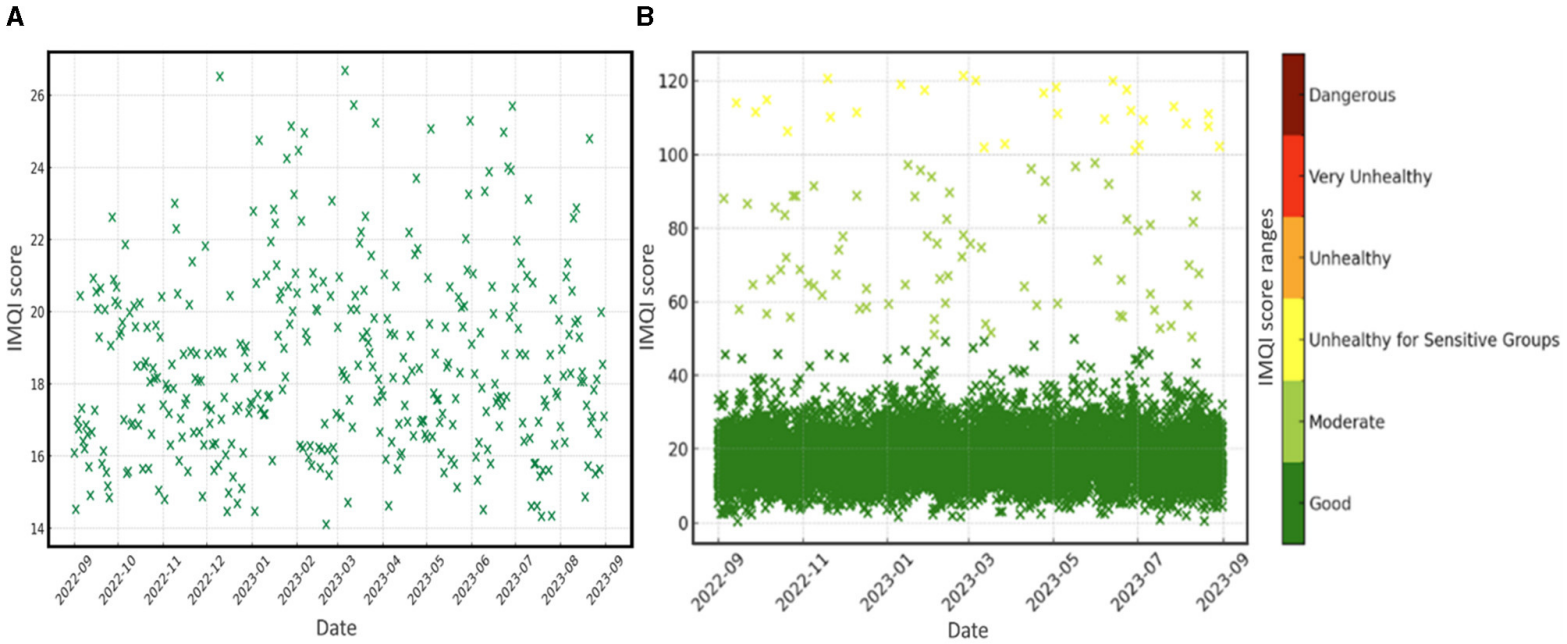
IMQI scores were obtained based on daily **(A)** and hourly **(B)** values of pollutants determined in the classroom.

The final IMQI results considering the pollutant average was 13.4. Such a score indicates that the internal microclimate in the classroom is of good quality, presenting no danger to human health. Analyzing the IMQI index for each hour of the data series, it appears that the scores vary between a minimum of 0.3 and a maximum of 121.4. Thus, these values fall into three different categories in terms of the influence they can have on human health. The best represented score range is between 0 and 50, which is individualized by a weight of 98.8%, followed by the category located between 50.1 and 100 which has 0.8% and the category 100.1–150, with only 0.4% (Figure 7B).

Although some hourly values are above the threshold that defines the ideal, the daily IMQI values are much better. Thus, the results demonstrated that all daily IMQI scores fall within the range of 0–50, with an average of 18.5, a minimum of 14.1, and a maximum of 26.7 (Figure 7A). This consistent categorization indicates that the air quality throughout the period is of a high standard, posing minimal health risks to human health. Thus, staff and students experience minimal exposure to air pollutants, reducing the risk of pollution-related health issues.

### 3.3 Statistical analysis of probabilities

**Figure 8:**
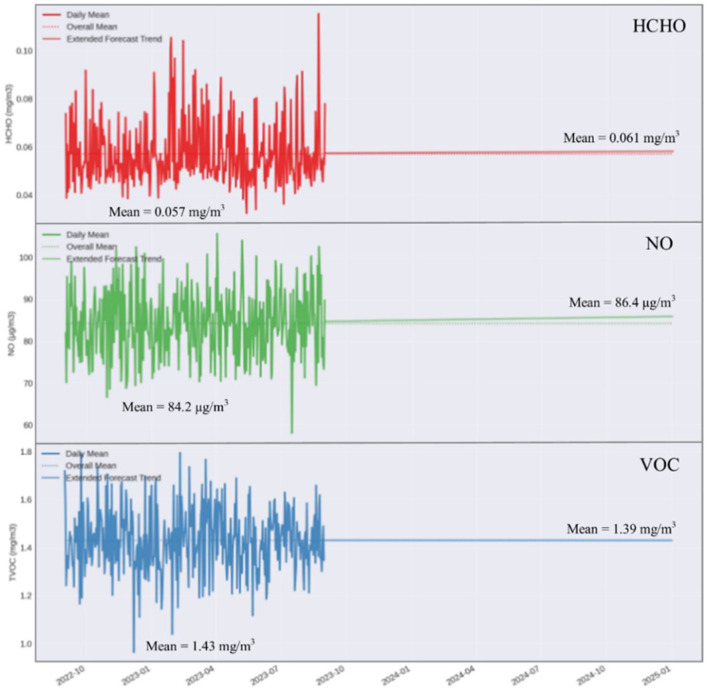
The results obtained after applying the ARIMA model for HCHO, NO, and VOC.

According to the ARIMA model applied to analyze and predict the evolution of pollutant levels in the classroom, different trends of evolution for pollutants until January 2025 (for the next year) were identified. The model revealed that the levels of CO_2_, CO, HCHO, TVOC, and O_2_ will show a stabilization, without significant variations in the near future. These results suggest that, in the absence of major changes in environmental conditions or classroom use, the concentrations of these pollutants will remain constant. On the other hand, the model showed that H_2_S and NO_2_ levels tend to decrease slightly. The reduction in the levels of these pollutants can be attributed to an improvement in ventilation and pollution source control measures toward the end of the monitoring period. In contrast, SO_2_ and NO concentrations tend to increase (Figure 8). The increase in SO_2_ and NO_2_ levels can be related to the use of certain materials or equipment that emit these pollutants, suggesting the need for interventions to reduce emissions. It is essential to understand the causes of these trends and take appropriate measures to ensure a healthy classroom environment. Continuous monitoring and adjustment of ventilation and pollution control strategies are vital to maintaining air quality within optimal parameters. These predictions emphasize the importance of continuous monitoring and proactive intervention to control and reduce air pollution in classrooms, thereby ensuring an environment conducive to the teaching-learning process and the health of all occupants.

**Figure 9:**
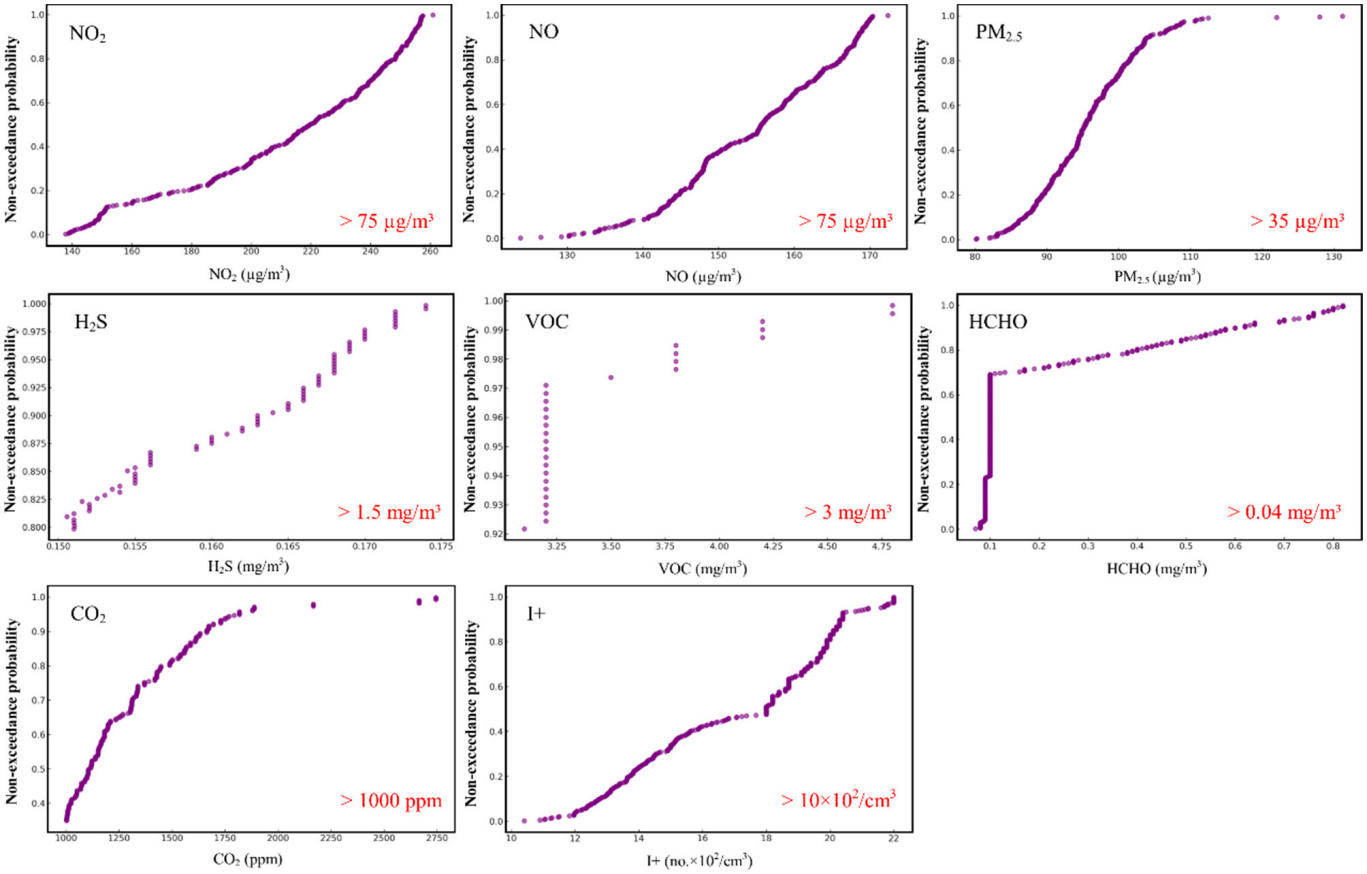
The empirical probability that the NO_2_, NO, PM_2.5_, H_2_S, VOC, HCHO, CO_2_, and I+ values exceed the limits imposed by the international standards in force.

In addition to creating models for predicting the evolution of indoor pollutant concentrations, it is crucial to determine the probability that these concentrations will exceed current international standards, thus indicating a potentially harmful environment for the health of staff and students. Thus, the EDF analysis indicates that HCHO is a pollutant that registers large and frequent exceedances of the international standards in force. The maximum values per day recorded exceeded 0.070 mg/m^3^ in all cases, the probability being very high that they will exceed the value of 0.1 mg/m^3^ (~76%-−0.237 EDF index). The probability that HCHO values exceed 0.5 mg/m^3^ is ~15% (0.848 EDF index), and that it exceeds 0.8 mg/m^3^ is 2% (0.979 EDF index). Regarding I+, the international standards in force, which show the maximum acceptability threshold as 1,000, were exceeded daily at least once. The EDF values for I+ indicate that a break above 1,400 is assigned an index of 0.23, while a value of 2,000 is assigned an EDF index of 0.809 and a high of 2,200 is assigned an EDF index of 0.998 (Figure 9).

A pollutant that recorded significant exceedances is PM_2.5_, for which the maximum daily measured values exceed 80 μq/m^3^ on all days. Thus, the value of 80.1 μq/m^3^ was given an EDF index of 0.002 (which indicates the fact that this value is very likely to be exceeded), the value of 100 μq/m^3^ has an index of 0.74, while 122 μq/m^3^ has a fairly low probability of exceeding, 0.993. As previously shown, both the NO_2_ and NO values have significantly exceeded the international standards that regulate the values of these pollutants at 75 μq/m^3^ in terms of daily variations. The obtained results indicate that in all the analyzed days, the values exceeded this limit at least once. The concentration twice the allowed limit was given an EDF index of 0.095 in the case of NO_2_ and 0.388 in the case of NO, and the value of 170 μq/m^3^ has an index of 0.185 in the case of NO_2_ and 0.979. At the same time, regarding NO_2_, the value of 200 μq/m^3^ was assigned an index of 0.333 and the value of 250 μq/m^3^, 0.837. H_2_S recorded maximum values above the allowed thresholds only in 74 days of monitoring. The probability that the values exceed the threshold of 0.15 mg/m^3^ is 20% (0.809 EDF index), and that the values exceed 0.170 mg/m^3^, the index rises to 0.968 (~3%). Regarding VOC, the allowed thresholds were exceeded in only 29 days by the maximum values of the pollutant. The probability that the values exceed 3.1 mg/m^3^ is only 7.8% (0.922 EDF index), and the probability that the values are at 4.2 mg/m^3^ is 1.3% (0.987 EDF index). In the case of CO_2_, values exceeding the standards of 1,000 ppm were recorded in 238 days. The EDF index for values exceeding 1,000 ppm is 0.349, for exceeding 1,500 is 0.818, while the index for them reacting to 2,167 ppm concentration is 0.974 (Figure 9).

EDF indices reveal significant and frequent exceedances of international standards for various pollutants, including HCHO, PM_2.5_, NO_2_, NO, H_2_S, VOC, and CO_2_. Given the direct impact on occupant health and comfort, these findings highlight the need for proper IAQ management.

The data also highlights several challenges and critical issues related to the indoor environment that require increased attention and appropriate action; of which it is essential to identify the specific sources of pollution and implement effective strategies to reduce them.

## 4 Discussion

The results obtained indicate that the pollutants that raise the greatest challenges for maintaining a healthy environment in the analyzed classroom are HCHO, VOC, PM_2.5_, NO, and NO_2_, due to concentrations above the thresholds allowed by the international standards in force.

Although the values of the five indicators registered some excesses, the question arises to what extent these exceedances have the characteristic of acting individually to induce health problems, even if all the other indicators are within normal limits. Some studies in the field support this, because it is well-known that HCHO is a respiratory irritant and is classified as a carcinogen, being associated with nasopharyngeal cancer and leukemia (77); PM_2.5_ can penetrate the lungs, causing cardiovascular and respiratory diseases, and is linked to increased mortality rates (78); NO_2_ is a respiratory irritant that can aggravate lung diseases and trigger asthma attacks (79); NO can contribute to the formation of ground-level ozone, which has negative health effects (80) and VOCs can cause headaches, dizziness, respiratory irritation and liver and kidney damage, some of which are carcinogenic (8).

Four of the five indicators that recorded high values (HCHO, VOC, NO, and NO_2_) behave independently of the time of day, showing large or small variations without being influenced by the time. In contrast, PM_2.5_ values (as well as PM_5_ and PM_10_) showed discontinuous maxima between 8 am and 8 pm from Monday to Friday, the period corresponding to the students' course schedule, suggesting the significant influence of human activity on these parameters (81, 82). In addition, human activity causes a slight increase in temperature (T) and a decrease in relative humidity (RH) in certain situations, as well as a considerable increase in CO_2_ levels and a slight decrease in O_2_ concentration. These exceeding of the allowed limits are supported by the specialized literature. A study by Ateş and Khameneh (83) demonstrated that classroom temperatures can exceed thermal comfort thresholds, and CO_2_ concentrations can reach alarming levels in the absence of adequate ventilation. Similarly, research by Kapalo et al. (84) highlighted a significant increase in CO_2_ levels during intense activities in classrooms, highlighting the crucial need for compliant HVAC systems.

In addition to identifying the pollutants that registered values above the permitted thresholds, a better understanding of the origin of these values is also of great importance. Thus, the evaluation of the way in which the five targeted pollutants interrelate and are determined by the other indoor pollutants through linear regression analysis (LR), indicated a limited ability to explain the variations in the levels of HCHO, VOC, PM_2.5_, NO_2_, and NO based-on variables. For the most part, the LP analysis shows that the influence of the large pollutants inside the classroom is quite small on the values of the indicators, even insignificant in some cases. The *R*^2^ levels are very low for each pollutant in relation to HCHO (average *R*^2^−0.057), VOC (average *R*^2^−0.059), and PM_2.5_ (average *R*^2^−0.082). NO had an average *R*^2^ coefficient of 0.3315, suggesting that the relationships between the other pollutants and NO are not very predictive and does not explain the model. At the same time, neither the values of other pollutants, such as O_3_, CO_2_, or NO_2_, do not significantly explain the variation in NO levels. The average *R*^2^ coefficient for NO_2_ is only 0.227, showing a modest predictive relationship with other pollutants such as O_3_, CO, and SO_2_. This suggests that while there are interactions between various atmospheric pollutants influenced by weather conditions and other external factors, the relationships are relatively weak.

It is well-known that the pollutants inside the classrooms are often influenced by external sources. The studies of Kaewrat et al. (85), Gaffin et al. (86), and Villanueva et al. (87) indicate that HCHO, VOC, PM_2.5_, NO, and NO_2_ values in classrooms can be significantly influenced by external sources, suggesting a strong correlation between the outside and the inside. In the case of the current study, based on the data obtained from the national air quality monitoring network, it was identified that there is also a weak correlation between PM_2.5_ concentrations outside and inside, with an *R*^2^ coefficient of ~0.36. This indicates a possible influence of outdoor pollutants on IAQ, suggesting that outdoor sources of PM_2.5_ may contribute to a certain extent to indoor levels, although the relationship is not very strong and does not explain much of the variation. In contrast, the influences of outdoor NO and NO_2_ on indoor levels are negligible, with correlation coefficients approaching zero. The authorized bodies of the Municipality of Oradea do not intend to determine the concentration of VOC or HCHO, therefore correlations could not be made between these outdoor and indoor indicators. But analyzing the links between other pollutants (O_3_, SO_2_, CO, etc.), the results also indicate weak correlations between outdoor and indoor concentrations. Therefore, we can only deduce the fact that the external concentrations of HCHO and VOC have a low influence on the interior too, especially since the classroom is at a significant distance from the main street; its influence being relatively low.

The aspects presented above indicate that the pollutants most likely have an internal source of emission (with a small exception regarding PM_2.5_). HCHO and VOC are often emitted by building materials and new furniture, as well as cleaning products and paints (69, 87). At the same time, the two can be emitted by electronic equipment, including computers. Studies show that certain synthetic materials and adhesives used in the manufacture of electronic equipment can emit these chemical substances (88). PM_2.5_ is determined by the activity of the students, who displace the sedimented particles inside and bring others from outside. High NO and NO_2_ values are often associated with unventilated heating sources, and studies indicate that NO and NO_2_ concentrations can be significantly higher in classrooms in modern buildings due to insufficient ventilation and emissions from internal sources (89).

Although the recorded values do not greatly and frequently exceed the standards in force and although students spend a limited amount of time indoors, pollutants can have a significant impact on students' wellbeing and performance. Studies show that continuous exposure to these chemicals can lead to fatigue and decreased ability to concentrate in students (69). They can also cause irritation of the eyes, nose and throat, headaches, and respiratory symptoms, exacerbate asthmatic symptoms in children (90), ultimately leading, according to some studies, to absenteeism (91).

Since the vast majority of pollutants most likely have internal sources, advanced occupancy monitoring and improved system operations could play a crucial role in managing indoor air quality. By integrating real-time occupancy sensors and their integration with HVAC systems, it is possible to optimize ventilation and air purification systems to respond dynamically to changes in room occupancy (92). This approach not only helps maintain pollutant levels within safe limits but also enhances energy efficiency by reducing unnecessary HVAC operation during low-occupancy periods (93). Additionally, investigating the specific activities or sources within classrooms that contribute to pollutant levels can help in developing targeted interventions. For example, reducing the use of certain materials or improving cleaning practices could mitigate the presence of allergens and other pollutants.

## 5 Limitation of the study

As in the case of any scientific approach, this study also presents some limits that must be considered for a correct interpretation of the results and to guide future research.

The first point concerns the limitations of LR and the ARIMA model. The LR values indicated a limited ability to explain the variability in HCHO, VOC, PM_2.5_, NO, and NO_2_ levels based on other variables within the classroom. Consequently, this suggests that there are likely other significant factors influencing these pollutant levels that were not accounted for in the model. The ARIMA model, although useful for short-term forecasting, can suffer from shortcomings related to unexpected changes in classroom use or environmental conditions that are not accounted for by the model. In the specific context of the characteristics of the building and the functionalization of the room analyzed, the quantification of the operating time of the HVAC system was impractical, considering its autonomous operation with respect to the presence of occupants. The occupancy rate varied significantly, which prevented rigorous record keeping. Additionally, the status of windows could not be quantified due to limited control over them. These aspects are complemented by other potential individual indicators, which, being independent of the authors, could not be effectively monitored.

Another critical aspect is the methodology for assigning weights to different indicators in the IMQI calculation. The weights assigned to the pollutants were established based on the literature, but this approach can be subjective and may vary depending on the sources used. At the same time, the influence of IAQ is quite relative and depends a lot on pre-existing conditions and the general condition of each individual.

At the same time, the use of standards (especially the US EPA for PM_2.5_ and PM_10_) and reporting basis (US EPA's IMQI scale) created for the outdoors in the indoor environment presents significant limitations due to the differences between the two environments. These standards do not consider the difference in exposure time, different pollution sources (building materials, furniture, and daily indoor activities), ventilation rates, and efficiency of air filtration systems, etc. But considering the fact that for certain pollutants and reporting scales there are no internationally accepted standards, or due to the fact that they are not enough research to represent a benchmark, in some studies it is necessary to use adapted thresholds. But on the other hand, the similar interior-exterior thresholds leave the possibility of a better comparative analysis.

## 6 Conclusions

Critical indicators for health, such as HCHO, VOC, PM_2.5_, NO, and NO_2_, frequently exceeded the limits allowed by the international standards in force, highlighting a significant environmental problem. Thus, HCHO concentrations exceed the daily international standards in force by 49.4%, VOC by 69.2%, PM_2.5_ by 99.5%, NO_2_ by 78.8%, and NO by 56.3%. All these parameters have a significant potential to harm human health. Although exceedances of international standards have also been recorded for other pollutants (T, RH, CO_2_, I+, I-, SO_2_, and PM_5_), these exceedances are sporadic and without a high harmful potential. It is worth noting that, despite these excesses, the limited time spent by students and teachers inside the classroom mitigates the impact of the internal microclimate on health, rather causing a decrease in concentration and the ability to perfect certain tasks.

Analysis of forecasts of pollutant levels for the coming year suggests the stabilization of CO_2_, CO, HCHO, TVOC, and O_2_ concentrations, while SO_2_ and NO levels could increase. It is essential to continuously monitor these pollutants to prevent exceeding the thresholds set by international standards. The highest EDFs are recorded for HCHO, PM_2.5_, NO_2_, and NO, underlining the need to implement adequate air quality control measures. The IMQI index was used to quantify indoor environmental effects on human health. The obtained values revealed that, although some of the pollutants are above the permitted limits, the general state of the microclimate was kept in the range of 0–50 in most cases, indicating the good quality of the microclimate and low health risks.

To improve IAQ it is crucial to improve ventilation systems and reduce pollution sources in the classroom by using less polluting building materials and furniture and implementing more efficient monitoring and air cleaning equipment. Future studies in this classroom will include assessing the perception of the students working here through a questionnaire, analyzing the bacterial and fungal microflora at the species and genus level, and correlating it with potential conditions determined. A direct relationship between IMQ quality and student academic performance will also be established.

## Data availability statement

The raw data supporting the conclusions of this article will be made available by the authors, without undue reservation.

## Author contributions

TC: Conceptualization, Formal analysis, Methodology, Supervision, Writing – original draft. AI: Data curation, Investigation, Validation, Writing – original draft. ZB: Funding acquisition, Project administration, Resources, Writing – original draft. HA-H: Software, Visualization, Writing – review & editing. DI: Data curation, Supervision, Writing – original draft. BS: Methodology, Writing – review & editing. TH: Investigation, Validation, Visualization, Writing – review & editing. GH: Conceptualization, Formal analysis, Writing – original draft. NH: Data curation, Investigation, Writing – review & editing. BB: Data curation, Software, Writing – review & editing. AP: Data curation, Writing – review & editing.

## References

[1] Zhang H-L Li B Shang J Wang W-W Zhao F-Y. Airborne pollutant removal effectiveness and hidden pollutant source identification of bionic ventilation systems: direct and inverse CFD demonstrations. Indoor Air. (2023) 20235522169. 10.1155/2023/5522169

[2] Van Tran V Park D Lee YC. Indoor air pollution, related human diseases, and recent trends in the control and improvement of indoor air quality. Int J Environ Res Publ Health. (2020) 17:2927. 10.3390/ijerph17082927 32340311 PMC7215772

[3] Ilieş DC Blaga L Hassan TH Ilieş A Caciora T Grama V. Indoor microclimate and microbiological risks in heritage buildings: a case study of the Neologic Sinagogue, Oradea, Romania. Buildings. (2023) 13:2277. 10.3390/buildings13092277

[4] Ilieş A Caciora T Marcu F Berdenov Z Ilieş G Safarov B. Analysis of the interior microclimate in art Nouveau heritage buildings for the protection of exhibits and human health. Int J Environ Res Public Health. (2022) 19:2416599. 10.3390/ijerph192416599 36554480 PMC9779619

[5] Marcu F Hodor N Indrie L Dejeu P Ilieş M Albu A. Microbiological, health and comfort aspects of indoor air quality in a Romanian Historical Wooden Church. Int J Environ Res Public Health. (2021) 18:189908. 10.3390/ijerph18189908 34574831 PMC8467041

[6] Saini J Dutta M Marques G. A comprehensive review on indoor air quality monitoring systems for enhanced public health. Sustain Environ Res. (2020) 30:1–12. 10.1186/s42834-020-0047-y PMC7400061 32659931

[7] Javid A Hamedian A Gharibi H Sowlat M. Towards the application of fuzzy logic for developing a novel indoor air quality index (FIAQI). Iran J Public Health. (2016) 45:203–13. 27114985 PMC4841875

[8] Vardoulakis S Giagloglou E Steinle S Davis A Sleeuwenhoek A Galea KS. Indoor exposure to selected air pollutants in the home environment: a systematic review. Int J Environ Res Public Health. (2020) 17:238972. 10.3390/ijerph17238972 33276576 PMC7729884

[9] Holden KA Lee AR Hawcutt DB Sinha IP. The impact of poor housing and indoor air quality on respiratory health in children. Breathe. (2023) 19:230058. 10.1183/20734735.0058-2023 37645022 PMC10461733

[10] Silva LBd Souza ELd Oliveira PAAd Andrade BJM. Implications of indoor air temperature variation on the health and performance of Brazilian students. Indoor Built Environ. (2020) 29:1374–85. 10.1177/1420326X1987822827409075

[11] Noda L Lima AV Souza J Leder S Quirino LM. Thermal and visual comfort of schoolchildren in air-conditioned classrooms in hot and humid climates. Build Environ. (2020) 182:107156. 10.1016/j.buildenv.2020.107156

[12] Wolkoff P Azuma K Carrer P. Health, work performance, and risk of infection in office-like environments: the role of indoor temperature, air humidity, and ventilation. Int J Hyg Environ Health. (2021) 233:113709. 10.1016/j.ijheh.2021.113709 33601136

[13] Pulimeno M Piscitelli P Colazzo S Colao A Miani A. Indoor air quality at school and students' performance: recommendations of the UNESCO Chair on Health Education and Sustainable Development & the Italian Society of Environmental Medicine (SIMA). Health Promot Perspect. (2020) 10:169–74. 10.34172/hpp.2020.29 32802752 PMC7420173

[14] Zhang L Morisaki H Wei Y Li Z Yang L Zhou Q. Characteristics of air pollutants inside and outside a primary school classroom in Beijing and respiratory health impact on children. Environ Pollut. (2019) 255:113147. 10.1016/j.envpol.2019.113147 31522002

[15] Brink H Loomans M Mobach M Kort H. Classrooms' indoor environmental conditions affecting the academic achievement of students and teachers in higher education: a systematic literature review. Indoor Air. (2020) 31:405–25. 10.1111/ina.12745 32969550 PMC7983931

[16] Jurado S Bankoff A Sánchez A. Indoor air quality in Brazilian universities. Int J Environ Res Public Health. (2014) 11:7081–93. 10.3390/ijerph110707081 25019268 PMC4113862

[17] Lee M Mui K Wong L Chan W Lee E Cheung C. Student learning performance and indoor environmental quality (IEQ) in air-conditioned university teaching rooms. Build Environ. (2012) 49:238–44. 10.1016/j.buildenv.2011.10.001

[18] Turunen M Toyinbo O Putus T Nevalainen A Shaughnessy R Haverinen-Shaughnessy U. Indoor environmental quality in school buildings, and the health and wellbeing of students. Int J Hyg Environ Health. (2014) 217:733–9. 10.1016/j.ijheh.2014.03.002 24709335

[19] Fromme H Diemer J Dietrich S Cyrys J Heinrich J Lang W. Chemical and morphological properties of particulate matter (PM_10_, PM_2.5_) in school classrooms and outdoor air. Atmos Environ. (2008) 42:6597–605. 10.1016/j.atmosenv.2008.04.047

[20] Becerra JA Lizana J Gil M Barrios-Padura Á Blondeau P Chacartegui R. Identification of potential indoor air pollutants in schools. J Clean Prod. (2020) 242:118420. 10.1016/j.jclepro.2019.118420

[21] Almeida S Canha N Silva A Freitas M Pegas P Alves C. Children exposure to atmospheric particles in indoor of Lisbon primary schools. Atmos Environ. (2011) 45:7594–9. 10.1016/j.atmosenv.2010.11.05233940420

[22] Bayati M Vu DC Vo PH Rogers E Park J Ho TL. Health risk assessment of volatile organic compounds at daycare facilities. Indoor Air. (2021) 31:977–88. 10.1111/ina.12801 33586827

[23] Lu KH Vu DC Nguyen QT Vo XT. Volatile organic compounds in primary schools in Ho Chi Minh City, Vietnam: characterization and health risk assessment. Atmosphere. (2021) 12:1421. 10.3390/atmos12111421

[24] Jin S Zhong L Zhang X Li X Li B Fang X. Indoor volatile organic compounds: concentration characteristics and health risk analysis on a university campus. Int J Environ Res Public Health. (2023) 20:5829. 10.3390/ijerph20105829 37239556 PMC10218183

[25] Bai L Dai H Wang J Li G. Source apportionment and health risk assessment of indoor volatile organic compounds. Indoor Built Environ. (2022) 31:1564–76. 10.1177/1420326X211065043

[26] Batterman S Grant-Alfieri A Seo S. Low level exposure to hydrogen sulfide: a review of emissions, community exposure, health effects, and exposure guidelines. Crit Rev Toxicol. (2023) 53:244–95. 10.1080/10408444.2023.2229925 37431804 PMC10395451

[27] Wigenstam E Elfsmark L Bucht A Jonasson S. Inhaled sulfur dioxide causes pulmonary and systemic inflammation leading to fibrotic respiratory disease in a rat model of chemical-induced lung injury. Toxicology. (2016) 368:28–36. 10.1016/j.tox.2016.08.018 27565714

[28] Song A Liao Q Li J Lin F Liu E Jiang X. Chronic exposure to sulfur dioxide enhances airway hyperresponsiveness only in ovalbumin-sensitized rats. Toxicol Lett. (2012) 214:320–7. 10.1016/j.toxlet.2012.09.010 23010223

[29] Nuvolone D Petri D Voller F. The effects of ozone on human health. Environ Sci Pollut Res. (2018) 25:8074–88. 10.1007/s11356-017-9239-3 28547375

[30] Ma Y Shen J Zhang Y Wang H Li H Cheng Y. Short-term effect of ambient ozone pollution on respiratory diseases in western China. Environ Geochem Health. (2022) 44:4129–40. 10.1007/s10653-021-01174-9 35001229

[31] Williams K Jones RJ Al-Rawi M. Particulate Matter (PM_2.5_) and mould characteristics in selected classrooms located in Waikato. N Zeal Prelimin Results Environ. (2023) 10:182. 10.3390/environments10100182

[32] Saad S Shakaff A Saad A Yusof A Andrew A Zakaria A. Development of indoor environmental index: air quality index and thermal comfort index. AIP Conf Proc. (2017) 1808:e020043. 10.1063/1.4975276

[33] Wagdi D Tarabieh K Zeid M. Indoor air quality index for preoccupancy assessment. Air Qual Atmos Health. (2018) 11:445–58. 10.1007/s11869-018-0551-y

[34] Dionova B Mohammed M Al-Zubaidi S Yusuf E. Environment indoor air quality assessment using fuzzy inference system. ICT Express. (2020) 6:185–94. 10.1016/j.icte.2020.05.007

[35] Environmental Indoor Air Quality Testing & Consulting. Indoor Air Quality (IAQ) Testing in Dallas Austin Houston. (2021). Available online at: https://emfsurvey.com/dallas-green-iaq-clearance-testing-post-construction-804-2-certificate-of-occupancy/ (accessed May 28, 2024).

[36] Environmental Protection Agency. Air Quality Index (AQI) Basics. (2018). Available online at: https://www.airnow.gov/aqi/aqi-basics/ (accessed July 29, 2024).

[37] Conceicao E Santiago C Lúcio M Awbi H. Predicting the air quality, thermal comfort and draught risk for a virtual classroom with desk-type personalized ventilation systems. Buildings. (2018) 8:35. 10.3390/buildings8020035

[38] Aydin K Yilmaz B. CFD-based multi-objective optimization of indoor air quality and thermal comfort in a classroom. Proc Inst Mech Eng E. (2023) 2023:e09544089231217960. 10.1177/09544089231217960

[39] Box GEP Jenkins GM Reinsel GC. Time Series Analysis: Forecasting and Control. 5th ed. Hoboken, NJ: Wiley (2015).

[40] Wei WWS. Time Series Analysis: Univariate and Multivariate Methods. 2nd ed. Boston, MA: Addison-Wesley (2006).

[41] Hamilton JD. Time Series Analysis. Princeton, NJ: Princeton University Press (2020).

[42] Hyndman RJ Athanasopoulos G. Forecasting: Principles and Practice. 2nd ed. Melbourne, VIC: OTexts (2018).

[43] American American Society of Heating Refrigerating and Air-Conditioning Engineers. ASHRAE Standard 55: Thermal Environmental Conditions for Human Occupancy. Atlanta, GA: American Society of Heating, Refrigerating and Air-Conditioning Engineers (2017).

[44] ISO 7730:2005. Ergonomics of the Thermal Environment—Analytical Determination and Interpretation of Thermal Comfort Using Calculation of the PMV and PPD Indices and Local Thermal Comfort Criteria.18045571

[45] Rajagopalan P Andamon M Woo J. Yearlong monitoring of indoor air quality and ventilation in school classrooms in Victoria, Australia. Archit Sci Rev. (2021) 65:1–13. 10.1080/00038628.2021.1988892

[46] United States Environmental Protection Agency. Indoor Air Quality (IAQ). Washington, DC: U.S. Environmental Protection Agency (2018).

[47] Health Canada. Residential Indoor Air Quality Guidelines for Formaldehyde (2005).

[48] American American Society of Heating Refrigerating and Air-Conditioning Engineers. ANSI/ASHRAE Standard 62.1-2010; Ventilation for Acceptable Indoor Air Quality. Atlanta, GA: American Society of Heating, Refrigerating and Air-Conditioning Engineers (2010). Available online at: https://www.ashrae.org/file%20library/doclib/public/200418145036_347.pdf (accessed May 28, 2024).

[49] European Union. Directive 2006/15/EC. Available online at: https://op.europa.eu/en/publication-detail/-/publication/dd498d0c-1837-4935-805c-1dee7de714c8 (accessed July 29, 2024).

[50] American American Society of Heating Refrigeration and Air Conditioning Engineers. ASHRAE Position Document on Indoor Carbon Dioxide. Atlanta, GA: American Society of Heating, Refrigeration and Air Conditioning Engineers (2022). Available online at: https://www.ashrae.org/file%20library/about/position%20documents/pd_indoorcarbondioxide_2022.pdf (accessed May 28, 2024).

[51] EPA Standard. Health Effects Notebook for Hazardous Air Pollutants, Formaldehyde-CAS 50-00-0. Available online at: https://www.epa.gov/sites/production/files/2016-09/documents/formaldehyde.pdf (accessed May 28, 2024).

[52] World Health Organization. WHO Guidelines for Indoor Air Quality: Selected Pollutants. Geneva: World Health Organization (2010). 23741784

[53] World Health Organization Regional Office for Europe. In Air Quality Guidelines for Europe. Copenhagen: WHO Regional Office Europe (2000).

[54] European Union. Directive 2000/39/EC. Available online at: https://osha.europa.eu/en/legislation/directives/directive-2000-39-ec-indicative-occupational-exposure-limit-values (accessed July 29, 2024).

[55] Renye W Chuan-Yuan D Gui-Liang X Hai-Yong W Zhijian Y Jun-Rong Z. Study on the effect of indoor air quality by negative air ion of plant. J Anhui Agricult Sci. (2014) 2014:9491–4.

[56] Jayaratne E Fatokun JF Morawska L. Air ion concentrations under overhead high-voltage transmission lines. Atmos Environ. (2008) 42:1846–56. 10.1016/j.atmosenv.2007.11.017

[57] Rossi E Pecorini I Iannelli R. Methane oxidation of residual landfill gas in a full-scale biofilter: human health risk assessment of volatile and malodours compound emissions. Environ Sci Pollut Res Int. (2021) 28:24419–31. 10.1007/s11356-020-08773-6 32307686

[58] Semple S Apsley A Wushishi A Smith J. Commentary: switching to biogas-what effect could it have on indoor air quality and human health? Biomass Bioenergy. (2014) 70:125–9. 10.1016/j.biombioe.2014.01.054

[59] EPA Standard. The National Ambient Air Quality Standards for Particulate Matter-Epa Retains Air Quality Standards for Particle Pollution (Particulate Matter): Fact Sheet. (2020). Available online at: https://www.epa.gov/sites/production/files/2020-04/documents/fact_sheet_pm_naaqs_proposal.pdf (accessed May 28, 2024).

[60] Lin X Liao Y Hao Y. The burden associated with ambient PM2.5 and meteorological factors in Guangzhou, China, 2012-2016: a generalized additive modeling of temporal years of life lost. Chemosphere. (2018) 212:705–14. 10.1016/j.chemosphere.2018.08.129 30179835

[61] Lee S Chang M. Indoor and outdoor air quality investigation at schools in Hong Kong. Chemosphere. (2000) 41:109–13. 10.1016/S0045-6535(99)00396-3 10819186

[62] Kampa M Castanas E. Human health effects of air pollution. Environ Pollut. (2008) 151:362–7. 10.1016/j.envpol.2007.06.012 17646040

[63] Fiordelisi A Piscitelli P Trimarco B Coscioni E Iaccarino G Sorriento D. The mechanisms of air pollution and particulate matter in cardiovascular diseases. Heart Fail Rev. (2017) 22:337–47. 10.1007/s10741-017-9606-7 28303426

[64] Manisalidis I Stavropoulou E Stavropoulos A Bezirtzoglou E. Environmental and health impacts of air pollution: a review. Front Publ Health. (2020) 8:14. 10.3389/fpubh.2020.00014 32154200 PMC7044178

[65] Chen T-M Gokhale J Shofer S Kuschner WG. Outdoor air pollution: nitrogen dioxide, sulfur dioxide, and carbon monoxide health effects. Am J Med Sci. (2007) 333:249–56. 10.1097/MAJ.0b013e31803b900f 17435420

[66] Dv B. Adverse health impacts of air pollution–continuing problems. Scand J Work Environ Health. (1995) 21:405–11. 10.5271/sjweh.55 8824745

[67] Worton DR. Future adoption of direct measurement techniques for regulatory measurements of nitrogen dioxide: drivers and challenges. Environ Sci Technol. (2020) 54:14785–6. 10.1021/acs.est.0c04709 33169991

[68] Li H Wu J Wang A Li X Chen S Wang T. Effects of ambient carbon monoxide on daily hospitalizations for cardiovascular disease: a time-stratified case-crossover study of 460,938 cases in Beijing, China from 2013 to 2017. Environ Health. (2018) 17:82. 10.1186/s12940-018-0429-3 30477579 PMC6258455

[69] Norbäck D Hashim J Hashim Z Ali F. Volatile organic compounds (VOC), formaldehyde and nitrogen dioxide (NO_2_) in schools in Johor Bahru, Malaysia. Sci Total Environ. (2017) 592:153–60. 10.1016/j.scitotenv.2017.02.215 28319702

[70] Farrugia G Szurszewski JH. Carbon monoxide, hydrogen sulfide, and nitric oxide as signaling molecules in the gastrointestinal tract. Gastroenterology. (2014) 147:303–13. 10.1053/j.gastro.2014.04.041 24798417 PMC4106980

[71] Azuma K Kagi N Yanagi U Osawa H. Effects of low-level inhalation exposure to carbon dioxide in indoor environments: a short review on human health and psychomotor performance. Environ Int. (2018) 121:51–6. 10.1016/j.envint.2018.08.059 30172928

[72] Sarofim M Waldhoff S Anenberg S. Valuing the ozone-related health benefits of methane emission controls. Environ Resour Econ. (2017) 66:45–63. 10.1007/s10640-015-9937-6

[73] Mittal K Jain A Bansal T Kumar P Mittal A. Oxygen therapy. J Pediatr Crit Care. (2018) 5:60–8. 10.21304/2018.0504.00413

[74] Freitas M Pacheco A Verburg T Wolterbeek H. Effect of particulate matter, atmospheric gases, temperature, and humidity on respiratory and circulatory diseases' trends in Lisbon, Portugal. Environ Monit Assess. (2010) 162:113–21. 10.1007/s10661-009-0780-5 19252992

[75] Bourotte C Curi-Amarante A Forti M Pereira L Braga A Lotufo P. Association between ionic composition of fine and coarse aerosol soluble fraction and peak expiratory flow of asthmatic patients in São Paulo city (Brazil). Atmos Environ. (2007) 41:2036–48. 10.1016/j.atmosenv.2006.11.004

[76] Buczyńska A Krata A Van Grieken R Brown AD Polezer G De Wael K. Composition of PM_2.5_ and PM_1_ on high and low pollution event days and its relation to indoor air quality in a home for the elderly. Sci Tot Environ. (2014) 490:134–43. 10.1016/j.scitotenv.2014.04.102 24852612

[77] Sega K Fugaš M Kalinić N. Indoor concentration levels of selected pollutants and household characteristics. J Expo Anal Environ Epidemiol. (1992) 24:477–85. 1483031

[78] Chen L Lippmann M. Effects of metals within ambient air particulate matter (PM) on human health. Inhal Toxicol. (2009) 21:1–31. 10.1080/08958370802105405 18803063

[79] Hesterberg T Bunn W McClellan R Hamade A Long C Valberg P. Critical review of the human data on short-term nitrogen dioxide (NO_2_) exposures: evidence for NO_2_ no-effect levels. Crit Rev Toxicol. (2009) 39:743–81. 10.3109/10408440903294945 19852560

[80] Nelson BS Stewart GJ Drysdale WS Newland MJ Vaughan AR Dunmore RE. In situo zone production is highly sensitive to volatile organic compounds in Delhi, India. Atmos Chem Phys. (2021) 21:13609–30. 10.5194/acp-21-13609-2021

[81] Wang T Du H Zhao Z Zhang J Zhou C. Impact of meteorological conditions and human activities on air quality during the COVID-19 lockdown in Northeast China. Front Environ Sci Eng China. (2022) 10:877268. 10.3389/fenvs.2022.87726837236582

[82] Yun G Yang C Ge S. Understanding anthropogenic PM_2.5_ concentrations and their drivers in China during 1998-2016. Int J Environ Res Publ Health. (2022) 20:695. 10.3390/ijerph20010695 36613014 PMC9819118

[83] Ateş E Khameneh ET. Effects of the number of people, temperature, relative humidity, and CO_2_ parameters on indoor air quality in higher education institution classrooms. Edelweiss Appl Sci Technol. (2023) 7:164–81. 10.55214/25768484.v7i2.406

[84] Kapalo P Mečiarová L Vilčeková S Burdová E Domnita F Bacotiu C. Investigation of CO_2_ production depending on physical activity of students. Int J Environ Health Res. (2018) 29:31–44. 10.1080/09603123.2018.1506570 30068229

[85] Kaewrat J Janta R Sichum S Kanabkaew T. Indoor air quality and human health risk assessment in the open-air classroom. Sustainability. (2021) 13:8302. 10.3390/su131583029151006

[86] Gaffin J Petty C Hauptman M Kang C Wolfson J Awad Y. Modeling indoor particulate exposures in inner-city school classrooms. J Exposure Sci Environ Epidemiol. (2017) 27:451–7. 10.1038/jes.2016.52 27599884 PMC5340641

[87] Villanueva F Tapia A Lara S Amo-Salas M. Indoor and outdoor air concentrations of volatile organic compounds and NO_2_ in schools of urban, industrial and rural areas in Central-Southern Spain. Sci Total Environ. (2018) 623:222–35. 10.1016/j.scitotenv.2017.11.274 29212055

[88] Jiang C Li D Zhang P Li J Wang J Yu J. Formaldehyde and volatile organic compound (VOC) emissions from particleboard: identification of odorous compounds and effects of heat treatment. Build Environ. (2017) 117:118–26. 10.1016/j.buildenv.2017.03.004

[89] Esplugues A Ballester F Estarlich M Llop S Fuentes V Mantilla E. Indoor and outdoor concentrations and determinants of NO_2_ in a cohort of 1-year-old children in Valencia, Spain. Indoor Air. (2010) 20:213–23. 10.1111/j.1600-0668.2010.00646.x 20408900

[90] Permaul P Gaffin JM Petty CR Baxi SN Lai PS Sheehan WJ. Obesity may enhance the adverse effects of NO_2_ exposure in urban schools on asthma symptoms in children. J Allergy Clin Immunol. (2020) 146:813–20.e2. 10.1016/j.jaci.2020.03.003 32197971 PMC7501199

[91] Mendell M Heath G. Do indoor pollutants and thermal conditions in schools influence student performance? A critical review of the literature. Indoor Air. (2005) 15:27–52. 10.1111/j.1600-0668.2004.00320.x 15660567

[92] Esrafilian-Najafabadi M Haghighat F. Occupancy-based HVAC control systems in buildings: a state-of-the-art review. Build Environ. (2021) 197:107810. 10.1016/j.buildenv.2021.10781036437647

[93] Rana R Kusy B Wall J Hu W. Novel activity classification and occupancy estimation methods for intelligent HVAC (heating, ventilation and air conditioning) systems. Energy. (2015) 93:245–55. 10.1016/j.energy.2015.09.002

